# Editorial: Genomics, proteomics and immunological signatures as diagnostic, predictive, and prognostic biomarkers in head and neck cancers

**DOI:** 10.3389/fimmu.2023.1122736

**Published:** 2023-01-25

**Authors:** Afsheen Raza, Mehdi Bourouba, Said Dermime

**Affiliations:** ^1^ National Centre for Cancer Care and Research, Hamad Medical Corporation, Doha, Qatar; ^2^ Translational Cancer Research Facility, Translational Research Institute, Hamad Medical Corporation, Doha, Qatar; ^3^ Faculty of Biological Sciences, University of Science and Technology, Houari Boumediene (USTHB), Bab Ezzouar, Algiers, Algeria; ^4^ College of Health and Life Sciences, Hamad Bin Khalifa University, Doha, Qatar

**Keywords:** head and neck cancers, nasopharyngeal carcinoma, proteomics signature, genomics/molecular markers, immunological markers, cytokines, and soluble mediators

## Introduction

Head and Neck Cancer (HNC) is the sixth most common cancer type accounting for approximately 900,000 cases and over 400,000 deaths annually (1). Heterogeneous malignancies involving the oral nasal cavities, paranasal sinuses, pharynx, larynx, and salivary glands are associated with HNC. The 5-year survival rate for HNC is 64% with median overall survival length ranging from 1.4 to 8.7 years, with glottic larynx cancer having the longest and hypopharynx cancer the shortest. The risk factors most frequently associated with head and neck cancer include smoking, alcohol consumption, diet, human papillomavirus (HPV) and Epstein-Barr virus (EBV) infections. The North African and South-East Asian populations are the most affected by these types of cancers. Recent estimates predict a major increase from 45%-77%, in the number of people who will be affected by these types of tumors in the next 20 years (2).

**Figure 1:**
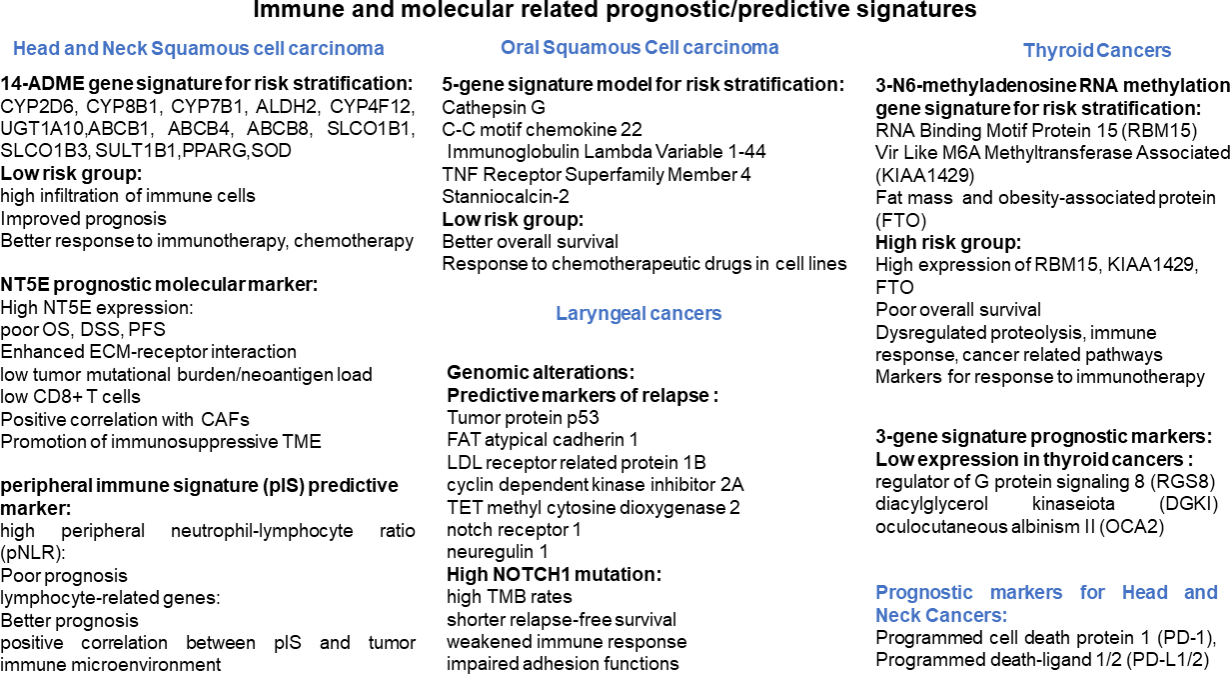
Schematic diagram showing signature biomarkers for Head and Neck Cancers.

Though there is a relatively increased understanding on the key factors leading to head and neck cancers, several studies have emphasized on the role of early diagnostic, predictive and prognostic biomarkers for improving survival rates in HNC patients (3–6). Recent advances in technologies have helped to understand molecular, metabolic, genetic and proteomic landscape of HNC. In addition to this, the utility of soluble mediators, cytokines, chemokines, soluble immune checkpoint and Natural killer cell markers are considered as non-invasive biomarkers that can be particularly helpful as an alternate to tissue biopsies. Furthermore, combination of these biomarkers with currently available diagnostic/screening tools could help in assessing risk and predicting response/progression. The Research Topic tilted *Genomics, Proteomics and Immunological signatures as diagnostic, predictive, and prognostic biomarkers in Head and Neck Cancers* aimed to shed light on this aspect in which 8 articles were published. These articles mainly focused on the molecular, metabolic, and genetic signatures of The Cancer Genome Atlas (TCGA) data sets of HNC. In this editorial, we have compiled a summary of these published articles to recap the important points addressed in the topic (**Figure 1**).

## Immune related prognostic and predictive signatures in head and neck squamous cell carcinoma

Metabolic, molecular, and immune-related signatures as predictive and prognostic biomarkers in head and neck cancers have unprecedented utility in patient management and survival. Drug metabolizing ADME (Absorption, Distribution, Metabolism, and Excretion) has been previously been explored as markers of therapy response, adverse drug reactions, drug resistance and survival outcomes. In lieu of this, Tang et al. identified 14 ADME genes using TCGA-HNSC data sets including phase I drug-metabolizing enzymes CYP2D6, CYP8B1, CYP7B1, ALDH2, CYP4F12, phase II drug-metabolizing enzymes UGT1A10, transporters ABCB1, ABCB4, ABCB8, SLCO1B1, SLCO1B3, SULT1B1 and modifiers PPARG,SOD. Furthermore, high, and low risk groups based on progression free survival (PFS) and overall survival (OS) showed distinct immune cell infiltration characteristics indicating that ADME related genes could serve as a robust prognostic tool to estimate immune status of HNSCC patients. Similarly, the role of immune cell infiltrates within the tumor microenvironment (TME) as predictive and prognostic biomarkers was also explored by study by Chen et al. The role of NT5E (gene encoding ecto-5’-nucleotidase-CD73) as a novel immune suppressive prognostic molecular marker in HNSCC was analyzed *via* RNA-seq data from TCGA database. The authors observed that upregulated NT5E expression was associated with poor OS, Disease Specific Survival (DSS) and Progression free survival (PFI). Furthermore, high NT5E was associated with enhanced ECM-receptor interaction potentially promoting EMT and metastasis, low tumor mutational burden (TMB), neoantigen load, CD8+ T cells and positive correlation with cancer-associated fibroblasts (CAFs) indicating that high NT5E promotes immunosuppressive TME. *In-situ* multicolor immunofluorescence staining validated the role of NT5E as a novel predictive biomarker of immune suppressive environment in HNSCC. On the other hand, a study by Hu et al. explored the role of peripheral immune signature (pIS) as a predictor of immune suppressive environment and treatment response in 599 HNSCC patients. The study reported an inverse correlation between peripheral neutrophil-lymphocyte ratio (pNLR) and survival with high NLR associated with prediction of poor prognosis. Validation *via* RNA-seq data from 913 HNSCC cases confirmed the association of lymphocyte-related genes with good while neutrophil-related genes with poor prognosis. Moreover, analysis on 30 HNSCC patients validated this positive correlation between pIS and tumor immune microenvironment (TIME) indicating that pNLR is a feasible prognostic/predictive marker in HNSCC.

## Immune related prognostic signatures in oral squamous cell carcinoma

Chen et al. reported on immune-related alterations in a total of 329 TCGA Oral Squamous Cell carcinoma (OSCC) cohort. Two immunity groups; immunity-high and immunity low groups were identified based on the degree of infiltration of 29 different immune cell types. The immunological/stromal score and immune-related differential genes observed within these groups were used to generate a robust five signature prognostic model (based on survival status) including Cathepsin G (CTSG), C-C motif chemokine 22 (CCL22), Immunoglobulin Lambda Variable 1-44 (IGLV1-44), TNF Receptor Superfamily Member 4 (TNFRSF4) and Stanniocalcin-2 (STC2). All five signatures were associated with low-risk group and better overall survival. Moreover, the author’s verified the behavior of these genes by treating the OSCC cell lines with standard of care chemotherapeutic drugs thus validating the role of these genes as reliable markers of disease progression in OSCC.

## Immune related prognostic signatures in thyroid cancers

Xia et al. extracted clinical data and RNA expression profiles of thyroid cancer from the Cancer Genome Atlas-thyroid carcinoma (TCGA-THCA) to shed light on the role of N6-methyladenosine (m6A) RNA methylation on clinical prognosis, immune infiltration, and immunotherapy response. Gene expression profiling showed that 4 out of 16 genes including Heterogeneous nuclear ribonucleoproteins C1/C2 (HNRNPC), RNA Binding Motif Protein 15B (RBM15B), Insulin Like Growth Factor Binding Protein 2 (IGFBP2) and E74 Like ETS Transcription Factor 3 (ELF3) were up regulated, while the remaining 12 genes were down regulated in thyroid cancer tissues compared to normal tissues. Further analysis from clinical tissues showed that 3 genes, RNA Binding Motif Protein 15 (RBM15), Vir Like M6A Methyltransferase Associated (KIAA1429) and Fat mass and obesity-associated protein (FTO) were associated with unfavorable prognosis. Furthermore, the high and low-risk groups showed distinct differences in the proteolysis, immune response, and cancer related pathways. Validation with 20 immunotherapy drugs indicated that the proposed risk model can act as an independent marker for immunotherapy targets. Similarly, Bai et al. also aimed to identify key molecules associated with thyroid cancer *via* 10 pairs of THCA tissues and noncancerous tissues to identify genes associated with THCA development, prognosis and clinical significance. Three genes including regulator of G protein signaling 8 (RGS8), diacylglycerol kinaseiota (DGKI) and oculocutaneous albinism II (OCA2) were related to thyroid hormone production, peripheral downstream signal transduction effects and infiltration of immune cells indicating that the respective genes are promising prognostic molecular markers in thyroid cancers.

## Molecular predictive signatures in laryngeal cancers

In addition to TCGA data, a study by Gong et al. gave insight on molecular and immunological signatures as predictive biomarkers in early-stage laryngeal cancer patients. Tissue samples from 52 T1-2N0 laryngeal cancer patients were analyzed by sequencing and NanoString immuno-oncology targeted RNA sequencing to identify distinct genomic mutation spectrum associated with relapse in the respective patients. A total of 469 genomic alterations were detected in 211 distinct cancer-relevant genes. The most frequently mutated genes in the patient cohort included tumor protein p53 (TP53, 78.5%), FAT atypical cadherin 1 (FAT1, 26%), LDL receptor related protein 1B (LRP1B, 19%), cyclin dependent kinase inhibitor 2A (CDKN2A, 17%), TET methylcytosine dioxygenase 2 (TET2, 17%), notch receptor 1 (NOTCH1, 12%) and neuregulin 1 (NRG1, 12%). Interestingly, high NOTCH1 mutation with high TMB rates were independent genetic factors that were significantly associated with shorter relapse-free survival (RFS) in recurrent laryngeal cancer. Furthermore, transcriptome analysis showed that recurrent tumors with NOTCH1 mutation displayed upregulation of the cell cycle pathway, decreased B and T cells immune signature and tumor-infiltrating lymphocytes (TILs) score. Moreover, weakened immune response and impaired adhesion functions was observed in NOTCH1-mutant patients in relapsed laryngeal cancer cohort evidencing the role of NOTCH1 mutation in prediction of relapse in laryngeal patients.

## Immune checkpoint molecules programmed cell death protein 1, programmed death-ligand 1 and programmed death-ligand 2 as prognostic markers in HNC

In addition to these highly impactful data sets in various head and neck cancer types, a review article by Jiang et al. discussing the importance of PD-1, PD-L1 and PD-L2 as prognostic markers in HNC gave insightful information on these controversial markers (14). The authors presented evidence from literature on the expression of these markers in pre-cancerous lesions, sub-cellular, cellular and tissue level localization with respect to prognostic evaluation. The review concluded with various suggestions including the utility of PD-1/PD-L1 expression as a risk warning in precancerous lesions, prognostic utility of PD-L1 expression at different subcellular levels and need for establishing a new PD-L1 evaluation system based on variability in PD-L1, PD-L2 expression in early and late-stage HNC samples.

## Conclusion

The Research Topic compiled impactful data mainly from TCGA data sets of HNSCC, OSCC, thyroid and laryngeal cancer. Molecular and immune signatures of the tumor microenvironment were identified as prognostic and predictive biomarkers. Since most of the data was from validated TCGA data, the articles pave the way for larger studies in clinical trial settings to explore their role in longitudinal monitoring in large patient cohorts.

## Author contributions

AR, MB, and SD developed and designed the topic, selected authors, and reviewers, reviewed submitted articles and supported their publication. AR, MB, and SD wrote the editorial. All authors revised the manuscript and approved the submitted version.
